# Genes driving three-dimensional growth of immortalized cells and cancer

**DOI:** 10.1038/s41419-025-07719-5

**Published:** 2025-06-10

**Authors:** Mukta Basu, Jin-Fen Xiao, Saravana Kumar Kailasam Mani, Fangyuan Qu, Yongqi Lin, Jason Duex, Huihui Ye, Vanessa Neang, Dan Theodorescu

**Affiliations:** 1 Department of Urology, Cedars-Sinai Medical Center, Los Angeles, CA USA; 2 Department of Pathology and Laboratory Medicine, Cedars-Sinai Medical Center, Los Angeles, CA USA; 3 Present Address: Department of Genetics, The University of Texas MD Anderson Cancer Center, Houston, TX USA; 4 Present Address: Department of Urology, University of Arizona, Tucson, AZ USA; 5 Present Address: University of Arizona Comprehensive Cancer Center, Tucson, AZ USA

**Keywords:** Urological cancer, Cell growth

## Abstract

Unchecked growth in three-dimensions (3D) in culture is a key feature of immortalized cells on the path to malignant transformation and hence a potential target phenotype for prevention. Also, expression of genes driving this process, but not that of 2D growth, would likely be more specific to cancer development and their inhibition would be less toxic to normal cells, many of which can grow in 2D but rarely in 3D culture. To define such genes, we compared CRISPR depletion screens performed in HBLAK, a spontaneously immortalized, non-tumorigenic human urothelial cell line, grown in 2D to those in 3D. Using the CRISPR Bassik DTKP (drug target kinase phosphatase) deletion library targeting 2,333 genes, we identified 85 genes which were specifically lost in 3D cultures. Prioritizing hits to those associated with bladder cancer in patients provided us with a set of 11 genes. Only one gene, MAPK1 remained relevant if a human pan-cancer criteria was applied. Single gene in vitro validation confirmed that MAPK1 inhibition was specific to 3D growth. We also found that MAPK1 depletion led to significant growth reductions in human tumor xenografts in vivo. Inhibition of MAPK1 by Ulixertinib, an orally active MAPK1 inhibitor, led to human bladder cancer growth inhibition in both 3D in vitro and in vivo models. In summary, screening for genes specifically driving 3D growth in immortalized cells may provide targets for both prevention and early therapy in bladder and other cancers while potentially limiting therapeutic toxicity.

## Introduction

Cancer medicine is slowly moving from precision medicine, treating established disease, to that of precision prevention, where primary and secondary prevention strategies target specific risk factors critical for transformation [1, 2]. The stepwise acquisition of changes like immortalization that culminate in a malignant tumor includes the ability to grow in three-dimensions (3D) and form colonies, which many normal cells do not possess in normal cell culture conditions [3–6]. Genetic screening using RNA interference and CRISPR are powerful tools to identify such genes. However, the utility of these approaches has been limited by gene redundancy, off-target effects, and discovering bona fide therapeutic targets that have minimal toxicity to normal cells [7–14].

CRISPR screening conducted in cancer cell lines grown in two-dimensional (2D) conditions have revealed a number of genes related to critical cellular functions in various cancer types [15–18]. However, other investigations have revealed substantial differences in gene expression when cells are cultured in 2D versus the more biologically relevant 3D condition [19–21]. Here we build on these studies with a specific objective of defining genes which interrupt the ability of 3D growth, but not 2D growth, in the context of cellular immortality, one of the hallmarks of malignant transformation [22]. We applied the Bassik-laboratory developed DTKP library (Drug targets, kinase and phosphatases library), which standardizes the length of CRISPR guide RNAs (17-18 base pairs) [14, 23], to identify genes that are specific for 3D growth of HBLAK, a spontaneously immortalized, non-tumorigenic bladder cell line which is an effective model for identifying genes involved in early transformation [24].

Bladder cancer (BLCA) is an especially good model for this study since it is expected to double its incidence worldwide by 2040 [25, 26]. Furthermore, given its propensity for “field carcinogenesis” [27, 28], which is responsible for driving multiple recurrences in the bladder after treatment of the initial primary lesions, it is an ideal cancer for secondary prevention strategies [29]. In this study, we found that MAPK1 depletion led to significant growth reductions in 3D cultures of HBLAK cells but not in 2D cultures, as well as significant growth reductions in human tumor xenografts in vivo. Inhibition of MAPK1 by ulixertinib, an orally active ERK1/2 inhibitor, led to human bladder cancer growth inhibition in both 3D in vitro and in vivo models. These findings demonstrate the proof of principle that CRISPR screening for genes specifically driving 3D growth in immortalized cells may provide targets for both prevention and early therapy in both bladder and other cancers, while potentially limiting therapeutic toxicity.

## Materials and methods

### Cell culture and generation of stable cell lines

HBLAK was cultured using a standard procedure [24] with CnT Prime Epithelial Proliferation Medium (CellnTec Advanced Cell Systems; CnT-PR). The cells were maintained in a 37 °C incubator at 5% CO_2_. Two cancerous cell lines, UMUC3 and UMUC6 were included for further validation. These cells were cultured with established culture medium and environmental conditions. Routine monitoring for mycoplasma contamination was performed using the MycoAlert Mycoplasma Detection Kit from Lonza (LT07-318; PS#668612).

Lentiviral backbone constructs containing shRNA against MAPK1 gene were purchased from Sigma Technologies (SHCLNG #TRCN0000010040) having the target sequence of CAAAGTTCGAGTAGCTATCAA binding to the coding region of the desired gene, packed with 2nd generation virus packaging system, containing psPAX2 and pMD2.G envelope plasmid. TransIT^®^-Lenti Transfection Reagent (Mirus, #MIR6604) was mixed with the Opti-MEM and standard procedure was done, followed by puromycin selection.

### Large-scale 2D culture of HBLAK cells

HBLAK cell two-dimensional (2D) growth was maintained in Nunclon Delta bioassay dishes (Thermo Fisher Scientific, #166508). The HBLAK cell numbers were optimized to seed at a density of 7000 cells/cm^2^ in 80 ml media on a square dish, which allowed them to continuously grow for 7 days without the necessity of passaging. Culture media for HBLAK cells was replaced every 3 days.

### Large-scale 3D culture of HBLAK cells

For large scale 3D culture of HBLAK cells in ultra-low attachment settings, 15 cm tissue culture plates were pre-treated with 6 ml of 20 mg/ml polyHEMA (Sigma, #P3932) dissolved in 95% pure ethanol (Fisher Scientific, #BP28184). For 3D culture, HBLAK cells were maintained in CnT-PR media with 0.5% methylcellulose (Fisher Scientific, #M-352). To determine the optimal cell density for 3D culture of HBLAK cells for CRISPR screens, cells were seeded at the density from 7500 cells/cm^2^ to 45,000 cells/ cm^2^ to ultra-low attachment plates (Corning, #3473). To monitor the viability in 3D culture, cells cultured in CnT-PR media with methylcellulose were collected and diluted with equal volume of DPBS before centrifugation. Cells were treated with accumax for 5 min before being counted with trypan blue (Gibco, #15250061). For long term 3D culture in this study, media was replaced at 0.2 ml/cm^2^ every two days. For CRISPR screening, a cell density seeding at 15,000 cells/ cm^2^ was selected, as a stable cell viability was obtained at this density of seeding. Media was replaced at 0.2 ml/cm^2^ every three days. 3D culture was collected after 7 days or 14 days of culture.

### CRISPR/Cas9 library screen with 2D and 3D cell growth

CRISPR-Cas9 depletion screen was conducted with Bassik Human CRISPR deletion library-Drug targets, kinases, and phosphatases (DTKP, Addgene, #101927) [14] using HBLAK cells. This library typically contains 24,569 sgRNA that targets 2,333 genes, hence having 10 guides for each gene targeted, along with 750 each of unique non-targeting and unique safe guides. HBLAK cells stably expressing Cas9 endonuclease was generated by infecting HBLAK cells with lentiCRISPRv2 hygromycin (Addgene, #98291). Infection of lentiviral DTKP sgRNA library into Cas9 expressing HBLAK cells was performed as previously described [3]. Briefly, to achieve a minimum of 1000 cells per guide RNA, 90 million HBLAK-Cas9 cells were infected with lentiviral DTKP sgRNA library at MOI 0.3. Two days after infection, cells were selected with 0.5 µg/ml puromycin for two days and recovered with puromycin-free media for an additional three days before being plated at time = 0. Cells were plated in duplicate in cell culture dishes. Twenty-five million cells were used for each replicate. Cells were pelleted by centrifugation. To get rid of excessive methylcellulose in the 3D growth setting, the cell pellets were treated with Accutase (Innovative Cell Technologies, #AT104). Genomic DNA was purified with Blood & Cell Culture DNA Maxi Kit (Qiagen, #13362) according to the instructions. To amplify sgRNA cassettes from the genomic DNA for the library construction, all purified genomic DNA was used, and two rounds of PCR were carried out with Herculase II Fusion Polymerase (Agilent, #600679) as previously described [9, 14].

### CRISPR screening analysis

The FASTQ sequencing files were analyzed using a Bowtie short-read aligner. The sgRNAs with high confidence were aligned and the Basic Local Alignment Search Tool (BLAST) was used to identify gene names. The quality of these sgRNA were checked. Preferably, dropout sgRNAs were the genes which are cancer essential genes and were lost due to prolonged growth in 2D or 3D settings. The effective size of these sgRNA and their corresponding sgRNA phenotype scores was calculated as previously described and below in more detail [3]. Briefly, log_2_ fold enrichment of each sgRNA was analyzed using a MAGeCK computational tool where each sgRNA was given a read count. The groups compared for this analysis were as follows: D7 vs. D0 and D14 vs. D0 in 2D setting as well as 3D setting. The median log_2_ fold enrichment was normalized against all non-targeting and safe sgRNAs in each growth setting to account for systematic bias in screens. Consecutively, the sgRNAs were aligned with their respective genes. The genes were further selected based on their 3D exclusivity, log_2_ fold enrichment < −0.1, and minimum number of sgRNAs dropped out ≥ 3.

#### Calculation of phenotype scores

The phenotype scores of the sgRNA were calculated as described previously by Kyuho et al. [3]. For each sgRNA, normalized scores from two replicates at each time point and growth setting were calculated. Briefly, effective size of each sgRNAs (pZ) was calculated by their log_2_ fold enrichments, at day 7 or day 14 normalized against day 0 in both 2D and 3D settings, which were further subtracted from the median log_2_ fold enrichments of all the control sgRNAs (non-targeting and safe guides).

**Effect size of each sgRNA (pZ) =** log_2_ fold enrichment of each sgRNA – median log_2_ fold enrichment of control/safe sgRNAs

Moving forward, the median value of all the sgRNA [10] that target a particular gene gives us the actual effect size of a gene (**U**_**gene**_). Next, we evaluated the phenotype scores of each gene (T score) as previously described [3]. In comparison to the pZ score, the phenotype score (T score) is calculated by:

$${\bf{P}}{\bf{h}}{\bf{e}}{\bf{n}}{\bf{o}}{\bf{t}}{\bf{y}}{\bf{p}}{\bf{e}}\,{\bf{s}}{\bf{c}}{\bf{o}}{\bf{r}}{\bf{e}}({\bf{T}}{\bf{s}}{\bf{c}}{\bf{o}}{\bf{r}}{\bf{e}})=({{\bf{U}}}_{{\bf{g}}{\bf{e}}{\bf{n}}{\bf{e}}}{\textstyle \text{-}}{{\bf{U}}}_{{\bf{c}}{\bf{n}}{\bf{t}}{\bf{r}}{\bf{l}}})/\surd ({{\bf{S}}}_{{\bf{v}}{\bf{a}}{\bf{r}}}/{{\bf{N}}}_{\exp }+{{\bf{S}}}_{{\bf{v}}{\bf{a}}{\bf{r}}}/{{\bf{N}}}_{{\bf{c}}{\bf{t}}{\bf{r}}{\bf{l}}})$$

where U_gene_ is the median effect of all sgRNAs (pZ) for a given gene, U_ctrl_ is the median effect of all negative control sgRNAs (pZ), and Svar is Var_gene_ × (N_exp_ −1) + Var_ctrl_ × (N_ctrl_−1), where Var_gene_ is the variance of sgRNA effects (pZ) for a given gene, N_exp_ is the number of sgRNAs for a given gene and N_ctrl_ is the average number of sgRNAs per gene in a given screen. The absolute T score was plotted in a scatter plot where modulus of the real number is plotted against the U_gene_ of each target gene. Consecutively, the phenotype score was checked for essential genes at their sgRNA level, like RPL8 and RPL15 to check its effectiveness.

### Survival analysis of BLCA cohort

The survival data of the BLCA cohort was obtained from KM plotter which assembles several patient pools like GEO, EGA, TCGA, Metabric, Impact, and PubMed repositories, predicting a robust outlook about the prognostic effect of a gene. The genes with hazard ratio > 1 and *p* value < 0.05 were selected for further study.

### MetMap 500 metastasis prediction tool

The metastatic potential of the genes was checked in the MetMap database [30]. This database curates the metastatic potential of 503 barcoded cell lines, spanning 21 different types of solid tumors. All the cell lines were pooled into 1 pool, followed by intracardiac injection into the fifteen 8–10-week-old mice. The metastatic potential of the cell lines was next correlated with the genome wide CRISPR viability data (gene effect). The effect of each gene and its metastatic potential was represented in a linear regression model, where the negative regression line reflects positive association of the gene effect with its metastatic potential.

### GEO dataset; BLCA cohort analysis and their correlation with proliferation score

To understand the effect of the 3D specific genes on transformation to cancer, we analyzed an RNA microarray study, GSE3167, which includes microarray data from 9 normal bladder urothelium samples, 13 carcinoma-in-situ samples, and 13 muscle invasive bladder carcinoma samples and the normalized gene expression was compared in these groups of patients, having different clinical stages.

The genes were additionally checked for their involvement in the BLCA progression among the TCGA cohort, which contained tumors of higher grade only, and screened for the important genes having significance of probability (p) >0.05. Next, the proliferative index of the tumors was analyzed by correlating a previously published proliferation signature [31] with the TCGA samples, which were grouped based on its median MAPK1 expression into high and low categories.

### Human protein atlas and TCPA outcome analysis

For correlating survival of the TCGA patients with the protein expression of MAPK1, at first the expression level of MAPK1 across different cancer types was checked in Human Protein Atlas database (https://proteinatlas.org/). Next, we downloaded the pan-cancer MAPK1 expression profile from the TCPA database (https://tcpaportal.org/) and analyzed the overall survival of the patients (t = 60 months). In TCPA, sub-cohorts of the TCGA samples were used for reverse phase protein array analysis. The obtained data were analyzed through three levels: (1) staining with validated antibody; (2) analysis of the staining spots against their dilution factor in the SuperCurve R package; and lastly, (3) subtracting the median intensity of each protein across all samples and giving out a normalized value. The normalized value for each antibody for each patient was used to analyze MAPK1 protein level in pan-cancer analysis [32, 33]. Similarly, the protein expression of MAPK1 was later correlated with the survival of the TCGA patients, pan-cancer wide.

### Tissue microarray

A commercially available human bladder microarray slide was obtained from US Biomax (Catalog number BL807). Briefly, this microarray slide contains a total of eighty cores, with 60 bladder malignant cases and 14 adjacent normal tissue. The rest of the cores are cancer adjacent tissues containing muscle layers and one adrenal gland tissue, which are excluded from our study. Each core is 1.5 mm in diameter and 5 µm in thickness. The slide was stained with MAPK1 antibody (Origene, #TA500475) and counterstained with hematoxylin, following the standard protocol of antigen retrieval with citrate buffer of pH 6.0 [34].

### Analysis of tissue microarray

The staining pattern of MAPK1 was assessed in the human microarray slide using QuPath, by two independent observers and then consulted by a pathologist. The staining pattern of the MAPK1 in both cytoplasm and nucleus was first set as a threshold in normal urothelial tissues and then overlapped in the tumor tissues samples to calculate the staining intensity and density in each human bladder core. The H score of each sample was plotted in dot plot in GraphPad Prism v9.2.0.

### Analysis of DepMap dataset

The Avana dataset (v.18Q4) for MAPK1 was downloaded from the DepMap website, which is primarily a Broad Institute’s project of cancer dependency Map. The gene effect CERES score of each of the genes was plotted for either pan-cancers or BLCA in GraphPad Prism v9.2.0.

### RNA Isolation and quantitative real-time PCR

High quality RNA was isolated from cells grown in 2D or 3D growth settings using Qiagen RNeasy Plus Mini kit (Cat. No.: 74134) and reversed transcribed using Invitrogen Superscript TM III Reverse Transcriptase kit (Cat. No.: 18080044). These cDNAs were used for real time quantitative PCR using primers for respective genes. Primers used: MAPK1 (F): ACACCAACCTCTCGTACATCGG; MAPK1 (R): GGCAGTAGGTCTGGTGCTCAA; GAPDH (F): GTCTCCTCTGACTTCAACAGCG; GAPDH (R): ACCACCCTGTTGCTGTAGCCAA. The knockdown effect of the guide RNA on MAPK1 RNA expression was checked by quantitative real time PCR using SYBR™ Green Universal Master Mix (Applied Biosystems^TM^, # 43-091-55) and calculated based on previously published data [26].

### 2D and 3D growth for validation

For the 2D growth assay, HBLAK, UMUC3 and UMUC6 cells were plated in a 6-well plate starting at 5000 cells per well. The standard culture medium for each cell type was changed every 3 days. The growth was assessed for 14 days, and then cells were counted in each well. The viability of the cells was also checked using Trypan blue. For the 3D growth assay, ultra-low attachment 96-well plates were used (Corning, # CLS7007) and phenol red free MEM media supplemented with 10% FBS and 1 mM sodium pyruvate was used for these cancer cell lines. Highly sensitive fluorescence based CyQUANT™ Cell Proliferation kit (Invitrogen; #C7026) was used to quantify cell number and its proliferation in both 2D and 3D growth mediums [35].

The same 2D and 3D protocol was followed while performing the ulixertinib (obtained from SelleckChem, #S7854) experiment. The drug was dissolved in 100% DMSO and added once to each well at a final concentration of 10 µM, 30 µM, and 60 µM concentrations [36]. For the HBLAK cells, approximately 9000 cells were plated to form spheroids and treated with ulixertinib at the same doses as delivered to cancerous cells. The viability of the 2D growth with Ulixertinib was assessed using CellTiter 96® AQueous One Solution Cell Proliferation Assay (MTS) reagent (Promega; # G3580), where 20 μl of the reagent was added per 100 μl of the 2D cell culture wells. After 4 h of incubation at 37 °C, reading at 490 nm was recorded at each time. Each time point was correlated with its respective control cells, where Ulixertinib was not added. For the 3D growth assay with Ulixertinib, the above mentioned CyQUANT™ Cell Proliferation kit was used.

### Competitive growth assay

For the competitive growth assay, we took advantage of non-invasive, non-cytotoxic fluorescent lipophilic dyes, Vybrant^TM^ DiD cell labeling solution (Excitation of 644 nm; Thermo Fisher Scientific, # V22887) or Vybrant^TM^ DiL cell labeling solution (Excitation at 550 nm; Thermo Fisher Scientific, # V22885) which were transferred to daughter cells for up to several divisions by lateral movement, but not to the adjacent cells [37, 38]. Separately we stained the empty vector controls of UMUC3 with DiL lipophilic dye and the shMAPK1 engineered UMUC3 cells with DiD for approximately 2 h. The vector control and the shMAPK1 cells were then mixed in equal proportions, plated for 3D growth in ultra-low attachment plates and photographed at day 0, 7, and 14. The same protocol was followed for UMUC6 cells.

### Immunoblotting

To obtain whole-cell protein extracts, monolayers were grown to 70-80% confluency and approximately 10^7^ total cells and then subjected to lysis using standard Pierce^TM^ RIPA lysis buffer (Thermo Fisher Scientific; #89900) [39]. After lysis, the homogenate underwent centrifugation at 12,000 rpm for 15 min at 4 °C, yielding a supernatant that was quantified using Pierce^TM^ BCA reagent (Thermo Fisher Scientific, #23225), aliquoted, and stored at −80 °C until needed. These lysates were utilized for western blot of MAPK1 antibody (p-MAPK1/3 (Thr202/Tyr204; Cell Signaling Technologies #9101), following the conventional Bio-Rad SDS-PAGE protocol, in triplicates [39]. The band intensities were subsequently normalized against the loading control, GAPDH (Cell Signaling Technologies #5174), using ImageJ software. The raw images of all the western blots have been added in the last supplementary figure (Fig. S5).

### Xenotransplantation and analysis of subcutaneous tumor growth

We conducted in vivo experiments employing age-matched male FOXN -/- mice (8 weeks) from Jackson Laboratories (NU/J, Homozygous for Foxn1 . Strain: 002019), according to institute approved IACUC protocol. To investigate the tumorigenic potential of HBLAK, we subcutaneously injected cells over a range of 3 × 10^6^, 4 × 10^6^, or 5 × 10^6^ HBLAK cells into both flanks of the mice.

Similarly, the influence of MAPK1 knockdown on tumor growth was assessed by injecting either 5 × 10^6^ UMUC3-empty vector/UMUC3-shMAPK1 or UMUC6-empty vector/UMUC6-shMAPK1 cells into age-matched male FOXN−/− mice (8 weeks) (*n* = 5). For data reproducibility, a minimum of five mice were maintained in each group of experiment. Tumor development and progression were meticulously monitored in all studies by measuring tumor dimensions biweekly for a period of up to 60 days, with tumor volume calculated using the formula (*L* × *W*^2^)/2, where ‘*L*’ represents the largest diameter of the tumor and ‘*W*’ is the shortest. Mice were euthanized upon reaching a predetermined endpoint (1000 mm^3^) specified by the IACUC-approved protocol. All mice were included in the analysis.

#### Ulixertinib treatment

Approximately 5 × 10^6^ UMUC3 and UMUC6 cells were injected into the right flank of the FOXN−/− mice in separate cohorts. After the tumors reached ~100mm^3^ in size, the mice were given ulixertinib (dissolved in 10% DMSO and 90% corn oil) via oral gavage for five consecutive days at a dose of 100 mg/kg [40, 41]. The tumor growth was assessed following the same protocol as described above. Mice were randomized to different experimental groups based on separate cages provided by the institute.

### Statistical analysis

Statistical analyses were performed using Prism v9.2.0 (GraphPad Software Inc., La Jolla, CA, USA) with results from three independent experiments, where each has three technical replicates, unless otherwise specified. Error bars, visible in all figures, including Supplementary Information (SI) figures, represent the Standard Deviation (SD). Significance levels were determined using the two-tailed student’s t-test or one-way ANOVA with Dunnett’s correction, and p-values are denoted as follows: *p < 0.05; **p < 0.01; ***p < 0.001; ****p < 0.0001.

## Results

### 3D CRISPR screening in human immortalized urothelial cells

**Fig. 1:**
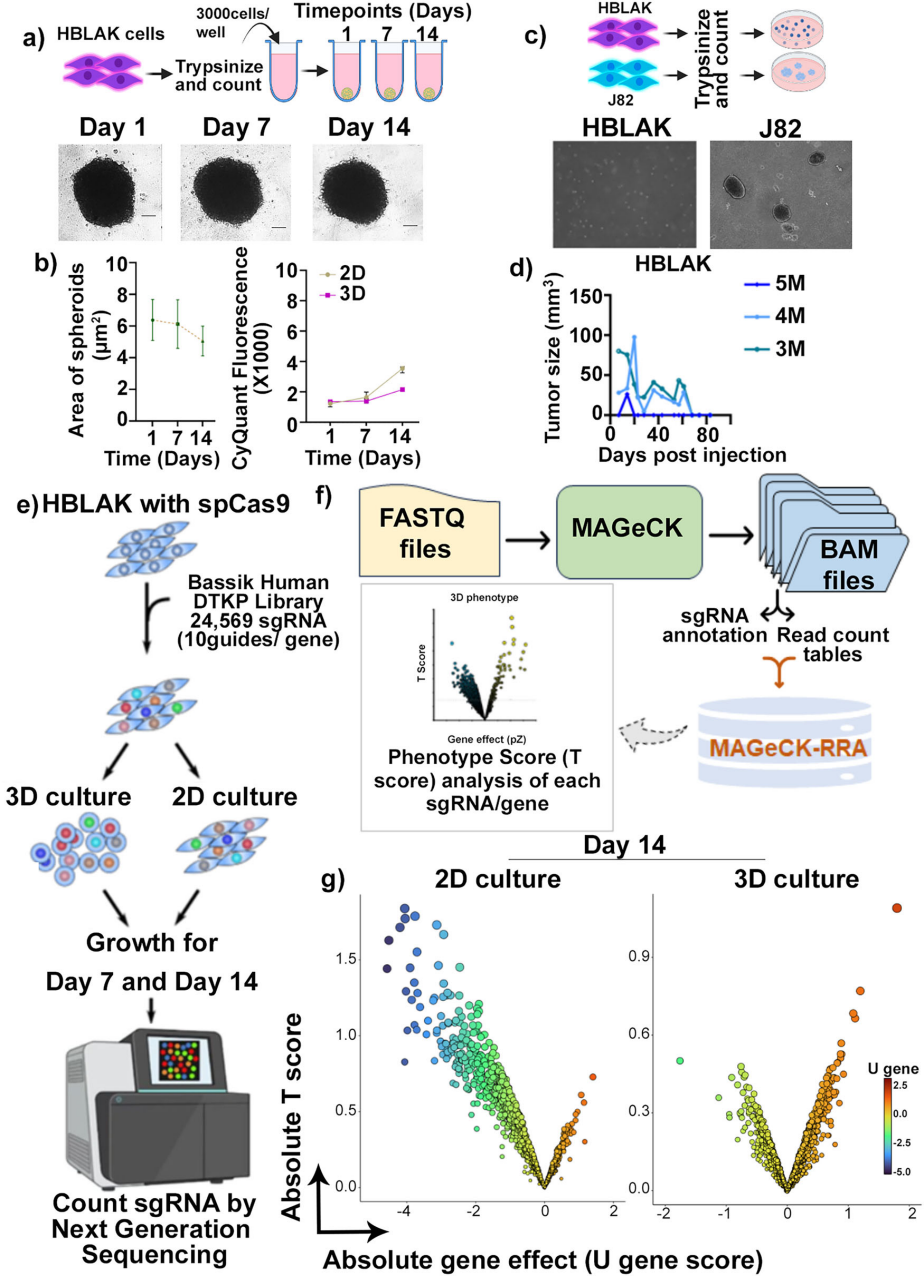
CRISPR screen in HBLAK cells: Workflow, data generation, and analysis. **a** Representative images of 3D growth of HBLAK cells in ultra-low attachment plates. The scale bar represents 100 µm. **b** Plots illustrating the area of spheroids and CyQuant fluorescence readings over time. **c** Images showing that HBLAK cells do not grow but survive in the soft agar clonogenic assay while J82 cells are able to grow and form viable colonies. **d** The growth kinetics of HBLAK xenografts in FOXN−/− mice when injected at different cell numbers, showing a gradual decrease and eventually disappearance of the xenografts. M: million. **e** Workflow of the CRISPR screening in HBLAK cells. The cells were first transduced with spCas9 containing virus, which were re-infected with the DTKP Bassik library. The positively transfected cells, selected via antibiotic, were allowed to grow in 2D and 3D growth conditions. The surviving cells were sequenced to identify the sgRNAs that dropped out over time. **f** Workflow depicting how the FASTQ files of sequencing data were processed using the MAGeCK method to produce a phenotype score for each gene. **g** Scatter plots showing the diverse phenotype patterns in the 2D—day 14 and 3D—day 14 normalized against day 0 at respective growth setting.

HBLAK is a spontaneously immortalized human bladder cell line that has the molecular features of early-stage urothelial tumors [24]. HBLAK cells formed 3D spheroids (Fig. 1a, b) but neither demonstrate anchorage independent growth in soft agar, in contrast to a well-known BLCA cell line, J82 (Fig. 1c) nor formed tumors in FOXN-/- mice (Fig. 1d). Given these characteristics, HBLAK cells were chosen for a CRISPR-based knockout screen to identify genes whose expression loss leads to cell death in either 2D and/or 3D growth conditions. The Bassik CRISPR DTKP library was transduced into HBLAK-Cas9 cells using lentivirus, and consecutively, these cells were plated in either 2D or 3D conditions. Cell aliquots were collected at different timepoints (day 0, day 7, and day 14) and then sequenced to determine the sgRNA counts at each of the time points (Fig. 1e]. Sequencing reads were used to generate a phenotype score as described above [3] (Fig. 1f). We observed significant differences in sgRNA counts and phenotype scores when sgRNAs were assessed on day 7 (Fig. S1) and day 14 compared to day 0, in both 2D and 3D growth conditions (Fig. 1g and Table S1).

### Genes lost with 3D-specific growth are associated with specific molecular pathways

**Fig. 2:**
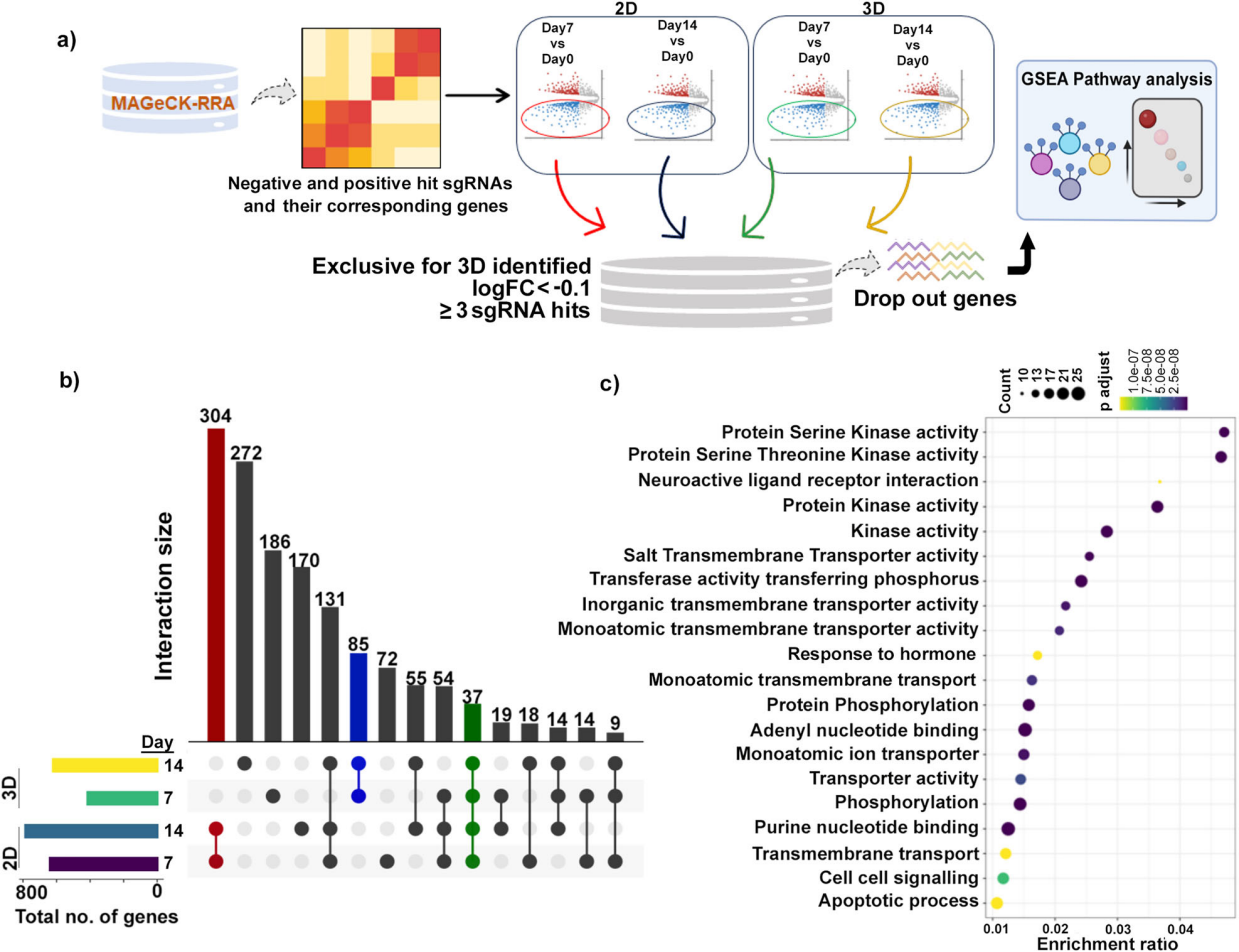
Identification of the 85 genes specifically dropped out in cells grown in 3D. **a** Schematic representation of how the FASTQ sequencing data have been processed to filter out the 3D exclusive genes, having the thresholds mentioned in the text. **b** The dropout genes from all the groups of analysis were placed in an UpSet plot to help identify the 3D exclusive genes common to both day 7 and day 14. The blue bar represents the 3D exclusive genes, the red bar represents the 2D exclusive genes, and the green bar represents genes common to both 2D and 3D. The bars on the left-hand side represent total number of genes in each group considered while creating the UpSet plot. **c** Overrepresentation analysis (ORA plot) involving the 85 3D exclusive genes shows the top 20 associated molecular pathways, where the size of the circle (Count) represents the number of genes affected in our study and the enrichment ratio represents the ratio of the count to the total number of genes reported in that pathway. The color gradient of the circle represents the statistical significance of the pathway.

The differential sgRNA counts suggested that different genes drop out as a function of different culture growth conditions. Using MAGeCK-RRA [42], genes at day 7 or day 14 were identified as enriched or dropped out when normalized to day 0. The dropout gene list for each growth condition was further filtered through thresholds, such as log Fold Change of < −0.1 and genes with ≥3 sgRNA hits (Fig. 2a). These dropped-out genes by day 7 and day 14, in both the 2D and 3D growth setting, were plotted in an UpSet plot (Fig. 2b and Table S2), to identify a unique set of 85 3D dependent genes, important for growth at both day 7 and 14 (Fig. 2b). Further, pathway analysis of these 85 genes revealed a significant enrichment for molecular functions such as kinase and transmembrane transporter activities, among others (Fig. 2c]. Interestingly, the 304 genes exclusive to 2D growth represented molecular pathways like dephosphorylation or cell cycle (Fig. S2). However, the essential 37 genes representing pathways common to both 2D and 3D growth revealed a circuit of common cellular maintenance molecular pathways like amino acid metabolism, tRNA aminoacylation, translation or oxidative phosphorylation (Fig. S2), signifying the efficacy of the filtering process and the model system.

### MAPK1 is a 3D growth essential gene in immortalized urothelial cells and poor prognostic factor in human bladder cancer

**Fig. 3:**
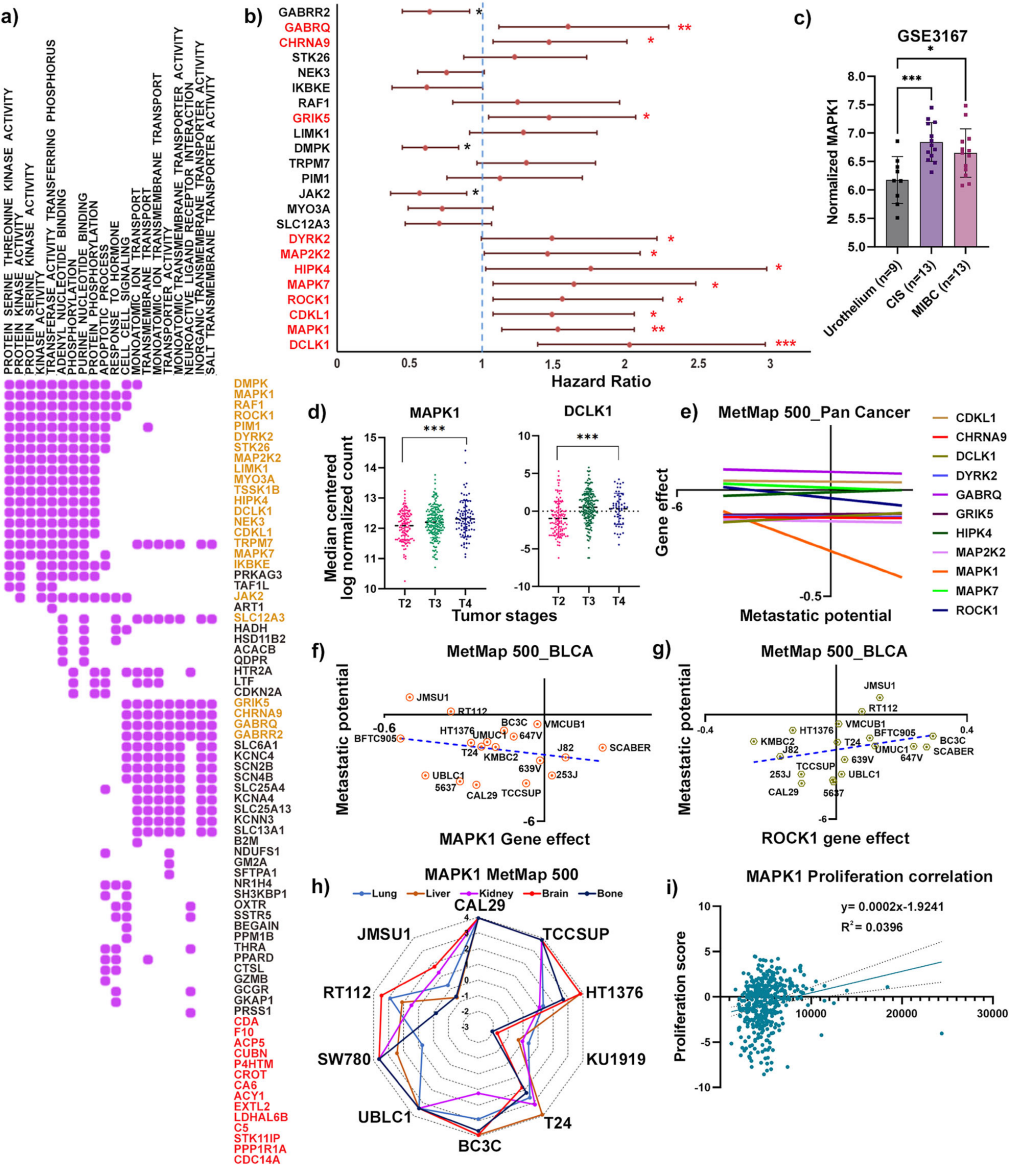
MAPK1 is a druggable gene whose expression is clinically relevant in multiple pan-cancer datasets. **a** The distribution of the 85 3D exclusive dropout genes in each of the top 20 molecular pathways from Fig. 2c shown as a Matrix plot, where each purple dot represents involvement of each gene in the molecular pathways. The 24 genes shown in yellow are involved in ≥9 of the 20 molecular pathways. The red font genes are the ones which were not involved in the top 20 molecular pathways, and hence excluded from this current study. **b** Forest plot of the 24 genes shows the hazard ratio (HR), as well as the p value, illustrating the overall survival of BLCA patients. The blue dotted line in the middle of the plot shows an HR of 1. The 11 genes with an HR > 1 and a *p* value < 0.05 have the p value highlighted in red with red asterisks and written in red fonts. Genes with HR < 1 but *p* value < 0.05 are marked with black asterisks and written with black fonts. **c** Bar graph showing normalized MAPK1 gene expression across the cell transformation stages among the patient pool in GSE3167 dataset. **d** The expression of MAPK1 and DCLK1 in the TCGA BLCA cohort were the only two genes to show an increase among the patients who had higher stages of BLCA. **e** The 11 genes were assessed in the pan-cancer MetMap 500 dataset which assess the metastatic potential of cell lines, and only MAPK1 and ROCK1 showed a positive correlation with metastatic potential. **f** In the MetMap 500 dataset concerning exclusively BLCA cell lines, MAPK1 shows a positive correlation with a negative slope, consistent with the pan cancer analysis. The correlation is represented by a blue dotted line, which is the best fitted regression line. **g** In the MetMap 500 dataset concerning exclusively BLCA lines, ROCK1 does not show a positive correlation between gene effect and metastatic potential, which means that depleting ROCK1 gene in the BLCA cell lines increases the metastatic potential of the cell lines. **h** Radar plot shows the common metastatic sites for these BLCA cell lines, where they are likely to metastasize. **i** The expression level of MAPK1 in cell lines is plotted against the proliferation signature score for each cell line, with green regression line showing a positive correlation between increased MAPK1 expression and increased proliferation.

The 85 3D exclusive dropout genes were next investigated for their involvement in the top molecular pathways (Fig. 2c), and we discovered 24 genes that were associated with at least 9 of the top 20 molecular pathways (Fig. 3a), implying the importance of this set of genes in regulating multiple important molecular signaling pathways. Therefore, next these 24 genes were investigated for their importance with disease-specific survival among TCGA-BLCA cohort. Interestingly, high expression of 11 of the 24 genes (MAPK7, MAPK1, MAP2K2, HIPK4, GRIK5, GABRQ, DYRK2, DCLK1, CHRNA9, CDKL1, ROCK1; hazard ratio >1) correlated significantly with poor prognosis among the patients (Fig. 3b). To further substantiate the vital role of these 11 genes across the tumors stages through normal urothelium, we analyzed a publicly available dataset, GSE3167, which is an RNA microarray dataset of 9 normal urothelium, 13 carcinoma-in-situ (CIS), and 13 MIBC samples. Analysis in this study illustrated that only MAPK1 (Fig. 3c) and GABRQ (Fig. S3a) RNA expression increased in both CIS and MIBC when compared to normal urothelium. However, among the other genes, only DYRK2 and ROCK1 showed increase in expression in CIS and MAP2K2 showed increased expression in the MIBC samples compared to the normal urothelium cohort (Fig. S3a). The remaining genes either showed no change in levels of gene expression or showed reduction of gene expression in the CIS or MIBC samples, as compared to the normal urothelium (Fig. S3a). A HIPK4 probe was not found in the microarray, so this screen hit was not included in this analysis. Furthermore, in TCGA-BLCA dataset, while investigating these 11 genes for their expression across tumor stage, only MAPK1 and DCLK1 showed significant increases in expression across stages (Figs. 3d and S3b). Moreover, of the 11 genes selected previously, only MAPK1 and ROCK1 showed positive correlation with metastatic potential in mice in the Pan-cancer MetMap 500 analysis (Fig. 3e). When looking exclusively at BLCA cell lines in mice, only MAPK1, not ROCK1, showed a significant negative correlation between gene expression and metastatic potential (Fig. 3f, g). More elaborately, lower the gene effect in DepMap reveals higher gene dependency of that cell line on MAPK1, was associated with reduced metastatic potential, implying an indirect correlation between MAPK1 depletion on reducing metastatic potential of the cell lines in mouse (Fig. 3f). When the metastatic potential of commonly used human BLCA cell lines was examined in MetMap, all revealed increased metastatic propensity apart from the KU1919 cell line (Fig. 3h). Interestingly, it was observed in the CCLE dataset that KU1919 had lower MAPK1 RNA expression in basal level than the other cell lines (Table S3), supporting the notion of a possible correlation between MAPK1 expression levels and metastatic potential of the cells. Next, we examined if expression of the 11 genes showed any correlation with proliferation index of a cell or tumor. Using a previously published proliferation signature consisting of MCM7, PLK1, MCM5, BUB1, MCM3, MKI67, MCM2, CCND3, MCM4, MYBL2, CCNB2, E2F1, MCM6, CCNE2, PCNA, CCND2, CCNB1, CCNE1, CCND1 genes, we found that only MAPK1 (Fig. 3i) and GARBQ (Fig. S3c) showed a significant positive correlation with proliferation score.

**Fig. 4:**
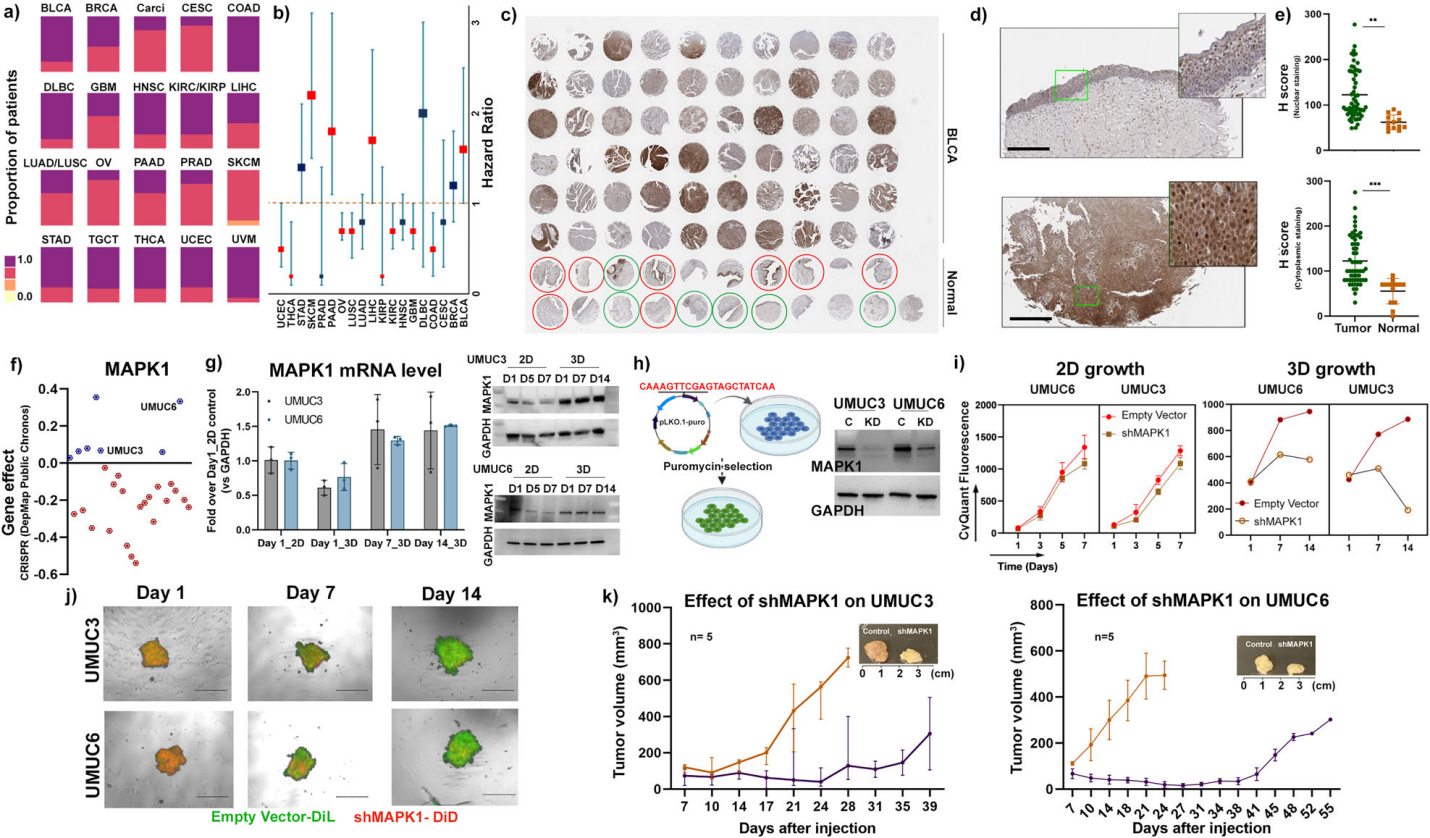
Expression of MAPK1 in BLCA patient tumor and its role in vitro and in vivo. **a** The bar plot represents the expression pattern of MAPK1 across cancer types where different color gradient depict different levels of MAPK1 expression. The cancer types were abbreviated as per TCGA guidelines (https://gdc.cancer.gov/resources-tcga-users/tcga-code-tables/tcga-study-abbreviations). **b** Forest plots depict the hazard ratio for pan-cancer patients, based on their MAPK1 protein expression. The brown dotted line in the middle of the plot indicates an HR of 1. The cancer types with significant p values are colored red. The size of the square boxes corresponds to the HR value. The red boxes depict having p < 0.05, while the blue boxes are non-significant. **c** Overall representation of a tissue microarray slide stained with MAPK1. The normal bladder tissues containing normal urothelial layer (as defined by the commercial provider) are circled in red, whereas the cancer adjacent tissues with normal urothelium (pathologically) are circled in green. The other cores (non-circled) contain muscle layers from normal tissues and are excluded from the quantitative analysis. **d** Enlarged representative images of a normal urothelium core sample (upper panel) and a cancer urothelium core sample (lower panel), highlighting that both cytoplasmic and nuclear staining of MAPK1 protein was observed. Scale bar = 90 µm. **e** Dot plots showing the H score of each cancer core and normal core in the TMA slide. The H score provides a cumulative factor undertaking both intensity and positive staining of MAPK1 in nucleus and cytoplasm individually. **f** Dot plot of all BLCA cell lines, curated in DepMap analysis and correlated with MAPK1 gene expression. Note the loss of MAPK1 has little negative impact on growth of some of the BLCA cell lines (blue dots) like UMUC3 and UMUC6, while loss of MAPK1 has a dramatic negative impact on growth (red dots <−0.2) in most BLCA cell lines. **g** mRNA and protein levels of MAPK1 gene in UMUC3 and UMUC6 cell lines in their 2D and 3D growth condition. **h** Graphical representation of knocking down MAPK1 in the cancer cell lines. The adjacent western blot reveals effective knockdown of the MAPK1 in the respective cell lines. **i** Growth curves of UMUC3 and UMUC6 in both 2D and 3D growth conditions, where the Y axis represents CyQuant fluorescence reading. **j** Microscopic images of spheroids obtained by mixing UMUC3_empty vector cells labeled with DiL dye (green) with UMUC3 shMAPK1 cells labeled with DiD dye (red) at a ratio of 1:1 at day 0 (top panels). The corresponding study in UMUC6 cells is shown in the bottom panels. By day 14, both UMUC6_empty vector and UMUC3_empty vector cells dominate the population. The images shown are merged images of fluorescent field and bright field. The scale bar is 3400 μm. **k** Growth curves of UMUC3 empty vector and UMUC3 and UMUC6 shMAPK1 xenografts in FOXN−/− mice (*n* = 5) show that both UMUC3 and UMUC6 shMAPK1 tumors grow significantly slower than empty vector control tumors. The inset picture shows representative tumors.

Following a strong gene association findings above, we next assessed the association of protein levels with human cancer using the Human Protein Atlas dataset [33]. When normal tissue was compared to their respective tumors types, we found that MAPK1 is significantly overexpressed not only in BLCA, but in most cancer types (Fig. 4a). Furthermore, the patient cohort in the TCPA dataset revealed that high MAPK1 expression is associated with poor survival in BLCA patients, as well as patients with liver hepatocellular carcinoma (LIHC), pancreatic adenocarcinoma (PAAD), and skin cutaneous melanoma (SKCM) (Fig. 4b). To potentially validate these findings, we performed an in-house staining on a human BLCA (*n* = 60) and normal urothelium (*n* = 14) tissue microarray slide with an anti-MAPK1 antibody. Staining was observed in both BLCA and normal tissue (Fig. 4c), and as expected in both the cytoplasm and nucleus (Fig. 4d). However, increased staining was found in tumor tissue relative to normal tissue (Fig. 4e), in both cytoplasmic and nuclear locations, validating the importance of elevated MAPK1 protein levels in the BLCA tumors over the normal urothelium.

### MAPK1 is a potential therapeutic target in human bladder cancer

Given that MAPK1 expression is associated with both bladder cancer formation as well as its prognosis, we sought to investigate the therapeutic potential of targeting MAPK1 in cancer. Using the DepMap portal, which investigates cancer vulnerabilities in human cancer cell lines in 2D growth setting, we found that MAPK1 depletion leads to cell death in most BLCA cell lines (Fig. 4f). However, since the DepMap data is based on 2D cell growth, we selected two cell lines out of the seven cell lines that were identified as not being sensitive to MAPK1 loss (UMUC3 and UMUC6) in the DepMap dataset. We checked both the mRNA and protein levels of MAPK1 in UMUC3 and UMUC6 cell lines under 2D and 3D growth conditions. Normalizing all data to day 1 of 2D growth, we found that at day 7 and day 14 of the 3D growth of UMUC3 and UMUC6 cells, the mRNA levels of MAPK1 increased about 50% (Fig. 4g). Interestingly, MAPK1 protein level at 3D conditions was already higher at day1 as compared to the 2D conditions and stayed consistent across day 7 and 14 in both UMUC3 and UMUC6, potentially due to the increased dependency on MAPK1 in 3D growth conditions (Fig. 4g). To further evaluate the significance of MAPK1 dependency in both these cells, we generated MAPK1 knockdown (MAPK1-KD) cells using lentiviral transduction in both UMUC3 and UMUC6 cells, and we assessed the growth of these MAPK1-KD cells in both 2D and 3D conditions (Figs. 4h and S3d**)**. Consistent with the DepMap data, MAPK1 depletion did not lead to any decrease in UMUC3 or UMUC6 growth in 2D conditions but did reduce growth in 3D conditions to a much greater extent (Fig. 4i). This 3D finding was further supported by observations made with a competitive growth assay, in which fluorescent lipophilic dyes are used for tracking cell proliferation [37, 38]. By day 14 in 3D growth conditions, UMUC3 or UMUC6 MAPK1-KD cells were outgrown by control UMUC3 or UMUC6 cells (Fig. 4j and S4a). Next, the MAPK1 depleted UMUC3 and UMUC6 cells, along with their empty vector controls, were injected into FOXN−/− mice (*n* = 5). Xenografts lacking MAPK1 expression grew significantly slower than control xenografts, leading also to a significant survival advantage to the knockdown mouse group (Figs. 4k and S4b).

### Pharmacologic inhibition of MAPK1 activity inhibits tumor growth

**Fig. 5:**
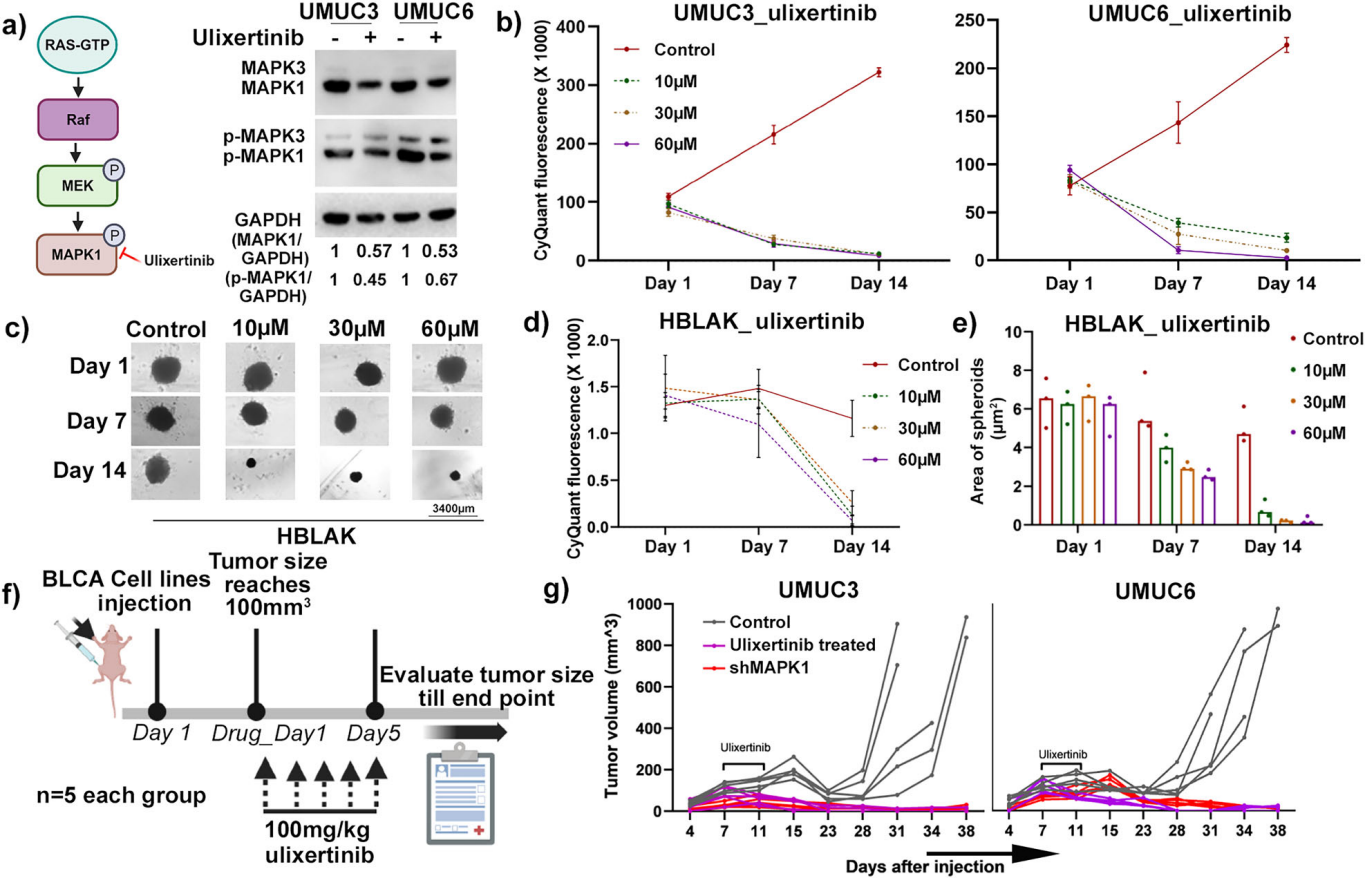
Pharmacologic inhibition of MAPK1 by ulixertinib reduces 3D growth in vitro and tumor growth in vivo. **a** Immunoblot showing treatment of UMUC3 and UMUC6 cells with the MAPK1 specific drug, ulixertinib, leads to decreased total MAPK1 protein, as well as decreased activated/phosphorylated MAPK1 protein (p-MAPK1), but no decrease on its homolog MAPK3. **b** Growth curves showing UMUC3 or UMUC6 cells treated with ulixertinib experience significant reduction in growth, relative DMSO treatment control, even at the lowest dose of drug. **c** HBLAK cell line treated with ulixertinib experiences significant reduction in 3D growth, compared to the DMSO treatment control, even at the lowest dose of drug, but at day 14. The scale bar is 3400 µm. **d** Line graph illustrating the reduction in growth of HBLAK cells after ulixertinib treatment at different timepoints. **e** HBLAK spheroid area, calculated from the spheroids shown in the previous figure (**c**) as well as the other biological replicates (not shown here), is plotted after ulixertinib treatment at different time points. **f** Schematic showing the xenograft generation and ulixertinib treatment strategy in mice (*n* = 5). **g** Growth curves of UMUC3 and UMUC6 xenografts with ulixertinib treatment/shMAPK1 resulted in dramatic reductions, and in some case complete disappearance of UMUC3 and UMUC6 tumors.

Ulixertinib is a small molecule inhibitor of MAPK1 [43]. To validate the effectiveness of ulixertinib on MAPK1 activity in UMUC3 and UMUC6 cells, we investigated MAPK1 protein levels and MAPK1 phosphorylation following ulixertinib treatment. Ulixertinib treatment reduced the expression level of active MAPK1, but not MAPK3 (Fig. 5a) in both cell lines, identifying the specificity of this drug. The effectiveness of ulixertinib on both 2D and 3D growth of HBLAK, UMUC3, and UMUC6 cell lines was assessed. All cell lines showed reduced growth only in 3D conditions, not in 2D, with ulixertinib treatment (Figs. 5b–e and S4c, d). However, to mention, UMUC3 and UMUC6 showed a significant response to ulixertinib treatment only at the highest dose (60 µM), under 2D growth conditions. Further, even at this highest drug dose, the cell viability did not drop lower than 50% by day 7, signifying the limited efficacy of the drug on the cancer cells at lower doses in 2D growth setting (Fig. S4d). To assess the efficacy of ulixertinib on in vivo growth, mice injected with either UMUC3 or UMUC6 xenograft tumors were orally treated with ulixertinib for 5 days (Fig. 5f) Ulixertinib had a dramatic impact, resulting in reduced growth of both UMUC3 and UMUC6 tumors (Fig. 5g). Even more exciting was the finding that tumor growth was minimal all the way to day 38, despite ulixertinib treatment ending on day 5. These ulixertinib treatment observations are consistent with the MAPK1-KD data, both in vitro and in vivo, and highlight the promising potential of the drug as a therapeutic in tumors with high expression of MAPK1. Additionally, ulixertinib may have a role in cancer prevention based on the sensitivity of non-tumorigenic HBLAK cells to MAPK1 loss and the long-term impact of ulixertinib treatment on tumor growth in mice, weeks after treatment ended. Thus, MAPK1 inhibition appears to have significant potential at inhibiting and preventing cancerous growth in BLCA patients and based on our findings from patient datasets in this study, potentially patients with other tumor types as well.

## Discussion

As scientific advances have accelerated our understanding behind the causes of cancer, there is also the realization that this same information provides opportunities for cancer prevention. The study presented here sought to screen an untransformed bladder cell line to identify vulnerabilities in these untransformed cells that could be exploited therapeutically to prevent carcinogenesis and potentially also to treat cancer. The study found that inhibiting MAPK1 in untransformed HBLAK bladder cells inhibited their in vitro 3D growth as well as reduced growth of cancer cells in vivo in mouse tumor models. These findings suggest that MAPK1 inhibition can dramatically limit growth of both a non-transformed bladder cells as well as bladder cancer (BLCA), supporting the idea that targeting MAPK1 can prevent transformation and treat established cancer.

One application for MAPK1 inhibition in the field of cancer prevention may be in older individuals who have been identified as having a high risk of developing BLCA. Given the uniqueness of BLCA, in that it can be screened non-invasively by analyzing voided urine, it is easier to identify high risk pre-cancerous patients than in many other cancer types. Thus, individuals can be non-invasively screened using health history (e.g., smokers) and analysis of voided urine to look for specific genetic alterations (e.g., MAPK1 pathway-related markers) which hint at field cancerization. Another population of individuals would include BLCA patients with treated non muscle invasive disease that are at high risk of recurrence and/or progression. BLCA patients whose tumors have higher expression of MAPK1, would be a particularly good patient population to consider.

Upon identification of MAPK1 as the most significant target in the screen and validating the finding by showing that MAPK1 inhibition via shRNA-based knockdown resulted in decreased cancer cell growth in vitro and in vivo, we looked for existing therapeutics which targeted the MAPK1 protein. The small molecule kinase inhibitor ulixertinib (BVD-523) has shown excellent promise in research studies to date [41]. In our study, it greatly inhibited 3D growth in the untransformed HBLAK screening line, as well as two bladder cancer cell lines. Furthermore, established UMUC3 and UMUC6 xenografts had their growth halted, and in some cases the tumors eliminated, with five doses of ulixertinib given orally. Thus, treatment of animals with the MAPK1 inhibitor ulixertinib provided further support that MAPK1 inhibition has strong potential as a prevention approach.

Even more encouraging has been the early success of ulixertinib in human clinical trials. Ulixertinib has been, or is being investigated, in seven Phase I and ten Phase II human clinical trials. These trials have included a variety of cancer types, most notably gastric and metastatic cancers like pancreatic, melanoma, or lymphoma [44–46]. This drug was also shown to have a dramatic effect on reverting multidrug resistance in some solid tumors [43]. Two clinical trials to date (NCT04566393, NCT02465060) have included BLCA patients among the study cohort but have not yet reported results. Significantly, in these studies the patient population is selected to those with MAPK pathway-altered tumors. Thus, genomic profiling of patient lesions will help increase the success rate of ulixertinib and minimize off-target effects for the patients.

Taken together, targeting MAPK1 in both pre-cancerous and cancerous cells leads to significant growth inhibition in pre-clinical models, and the MAPK1 inhibitory therapeutic ulixertinib appears to have a good safety profile and excellent efficacy in MAPK1 selected cancer patient populations. Our work provides a rationale for considering MAPK1 inhibition via ulixertinib as a potential prophylactic treatment for either primary or secondary BLCA prevention in high-risk populations, particularly those in the latter group exhibiting alterations in the MAPK pathway. While there are always early toxicity concerns when considering taking any agents for extended periods of time as a preventive strategy, ulixertinib seems to be well tolerated across all ages [47].

## Supplementary information

Supplementary information_word file

Table S1

Table S2

Table S3

## Data Availability

All data underlying the results in this article are available as part of the article and in the supplementary files. No additional source data are required.
