# Supplementary material for: Genes driving three-dimensional growth of immortalized cells and cancer
Source: Cell Death Dis. 2025 Jun 10;16(1):442. doi: 10.1038/s41419-025-07719-5 (PMC12152191; doi:10.1038/s41419-025-07719-5)
Supplement: Supplementary file 1 — Supplementary information_word file [file 41419_2025_7719_MOESM1_ESM.docx]

# **Supplementary data**

**Figure S1:** **Scatter plot showing phenotype scores of the sgRNAs found in Day 7 vs. Day 0 in both 2D and 3D growth conditions.**


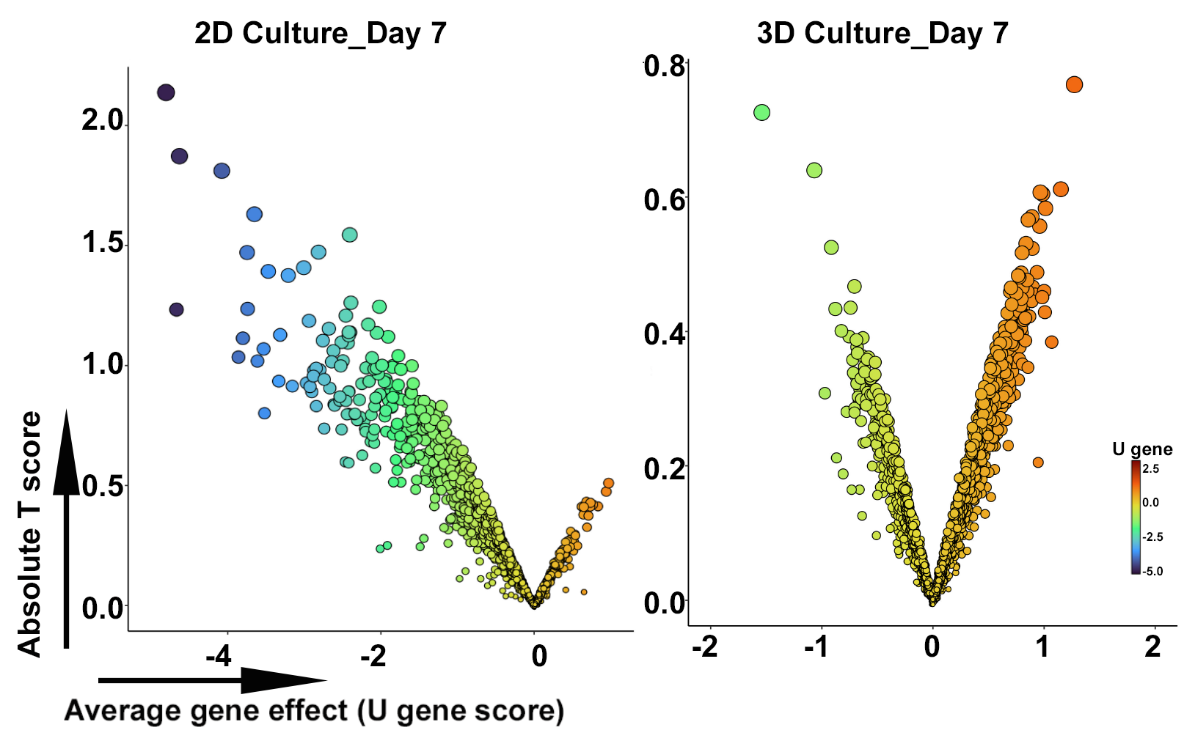


## **Figure S2: Molecular pathways altered with in vitro cell culture**


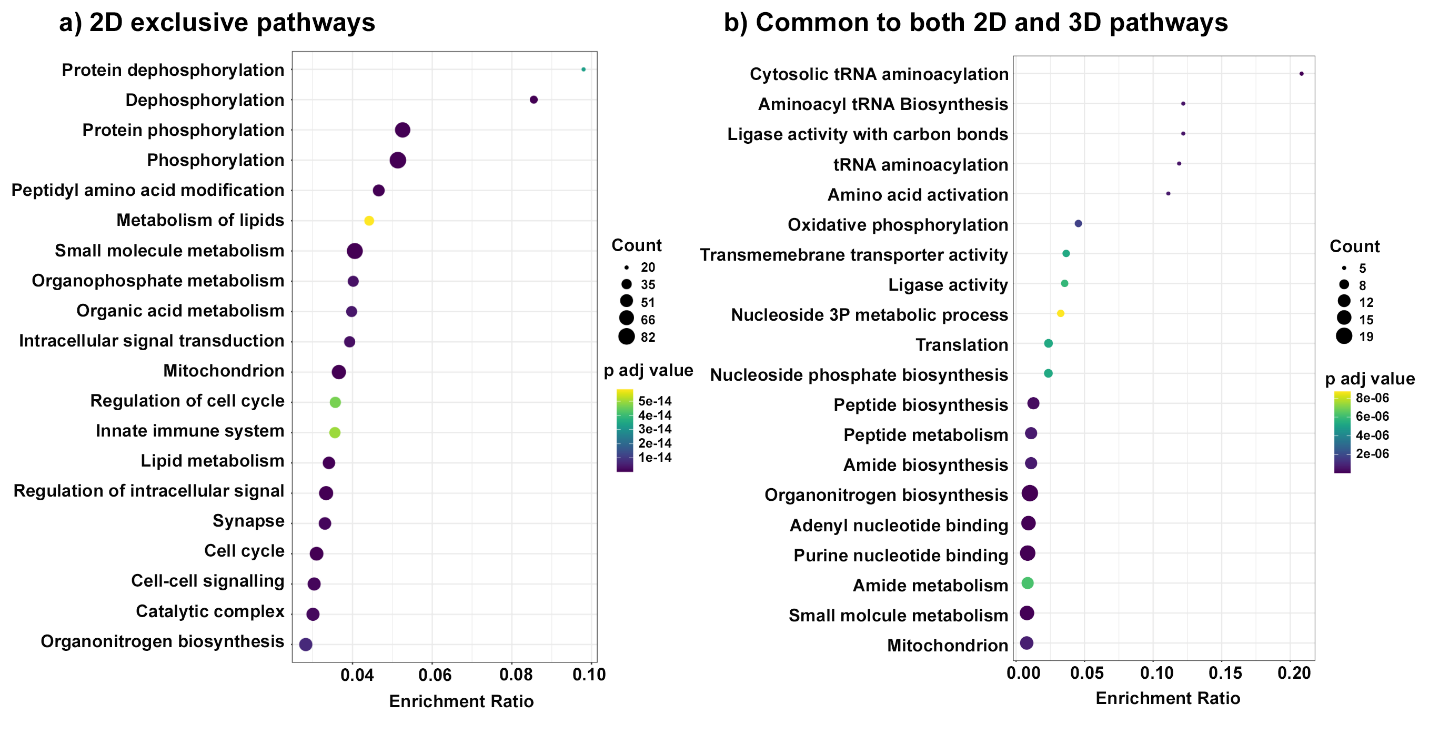


1. Molecular pathways involved exclusively in 2D growth conditions. Molecular pathways leading to phosphorylation or dephosphorylation of proteins are top hits.
2. Molecular pathways involved in both 2D and 3D growth conditions. Essential metabolic pathways are the top hits, such as amino acylation or RNA biogenesis.


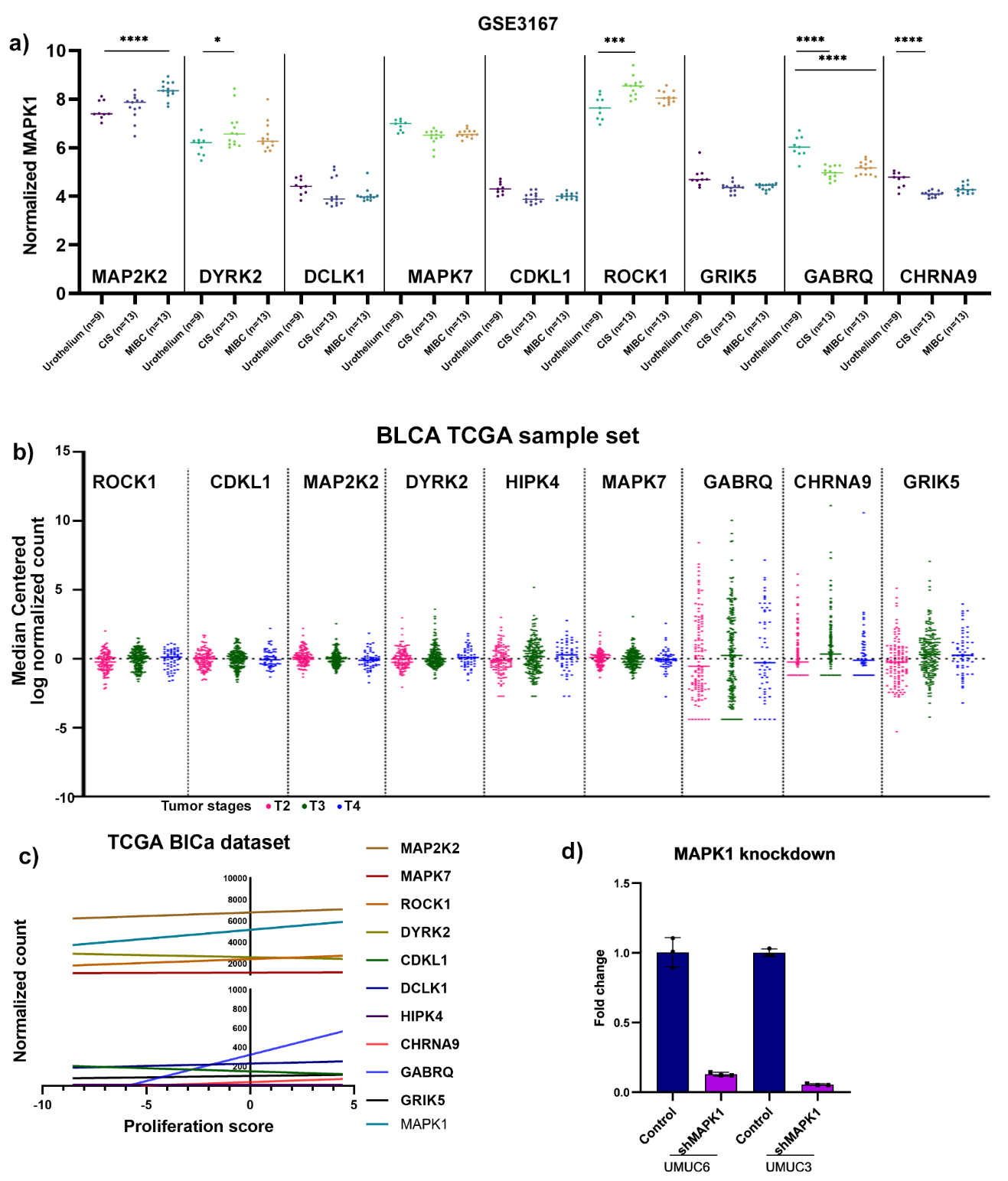
**Figure S3: Expression pattern of genes in TCGA**

1. Dot plot representing normalized gene expression across different samples in the GSE3167 dataset
2. Dot plot representing expression pattern of TCGA patients across stages for the 9 genes shown, with none of them showing any change in expression as the stage increases.
3. The 11 genes, sieved out from the KM plotter analysis, were correlated with the proliferation signature where only MAPK1 and GABRQ shows positive correlation.
4. Histogram showing effective knockdown of MAPK1 in UMUC3 and UMUC6 cells after lentiviral transduction of its corresponding sgRNA, analyzed by quantitative PCR.

##
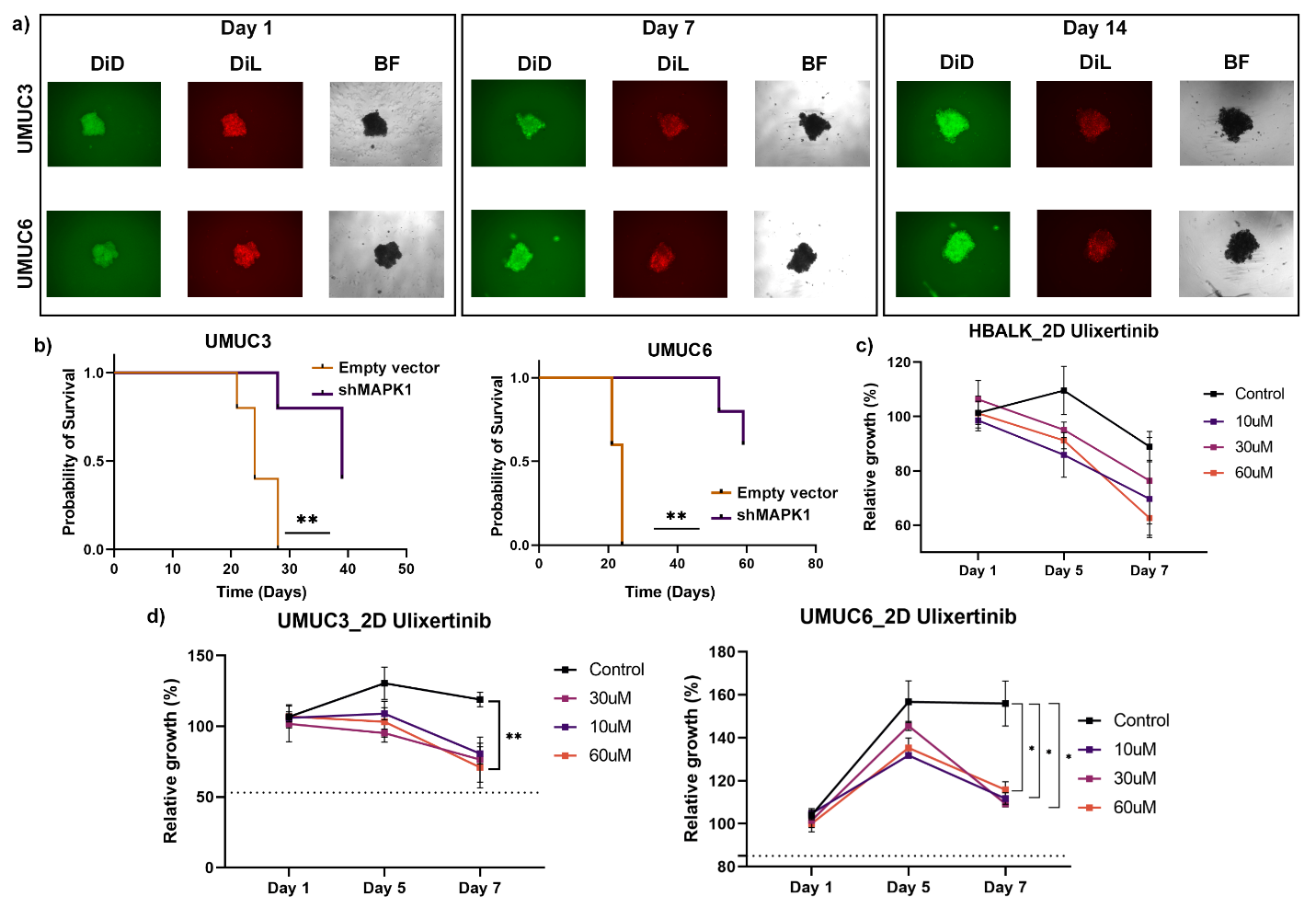
**Figure S4: Raw images and graphs of the 3D and 2D growth**

1. Individual microscopic fields of the merged images shown in Figure 4h. BF: Bright Field microscopic image.
2. Kaplan-Meier survival curves of mice harboring UMUC3_and UMUC6 shMAPK1 xenografts show significantly better survival than their respective empty vector controls.
3. Trend graph showing the growth rate of HBLAK cells under different doses of Ulixertinib treatment from day1 to day 7.
4. Trend graph showing the growth rate of UMUC3 and UMUC6 cells under different doses of Ulixertinib treatment from day1 to day 7.

**Figure 5: Raw images of the western blots used in this manuscript**


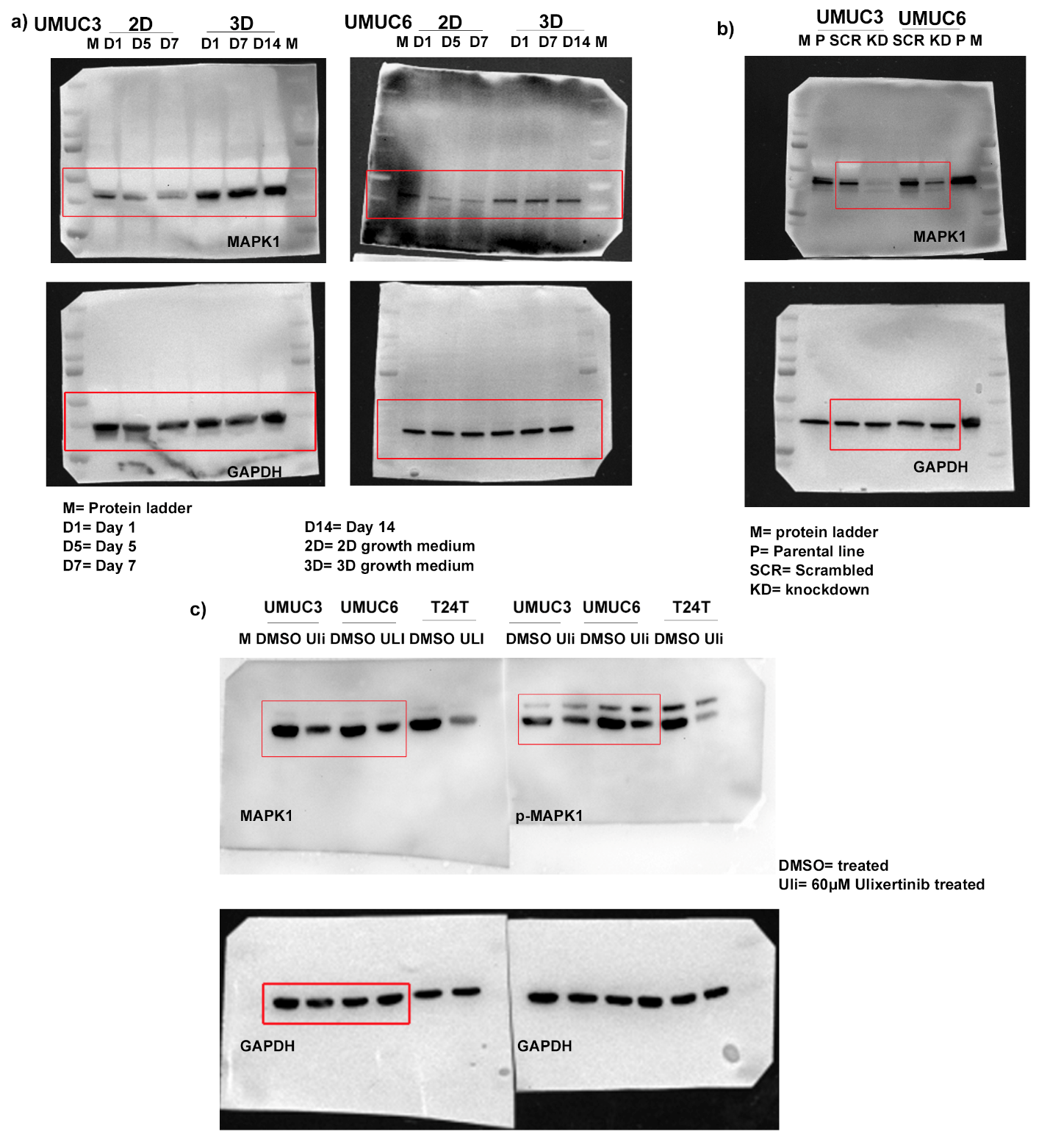


1. Raw images for the figure referred to figure 4g
2. Raw images for the figure referred to figure 4h
3. Raw images for the figures referred to figure 5a

**Table S1:** Table representing the raw results obtained from the MAGeCK computational tool after analyzing the HBLAK sequencing at different time points in different growth settings.

**Table S2:** Detailed information about the genes at different interaction sites of the UpSet plot shown in Figure 2b.

**Table S3:** Detailed information about the cell lines and their respective RNA expressions in the CCLE database and their reversed metastatic potential, as reported in the MetMap500.
